# High-resolution targeted bisulfite sequencing reveals blood cell type-specific DNA methylation patterns in *IL13* and *ORMDL3*

**DOI:** 10.1186/s13148-021-01093-7

**Published:** 2021-05-10

**Authors:** Cilla Söderhäll, Lovisa E. Reinius, Pertteli Salmenperä, Massimiliano Gentile, Nathalie Acevedo, Jon R. Konradsen, Björn Nordlund, Gunilla Hedlin, Annika Scheynius, Samuel Myllykangas, Juha Kere

**Affiliations:** 1 Department of Biosciences and Nutrition, Karolinska Institutet, Stockholm, Sweden; 2 Department of Women’s and Children’s Health, Karolinska Institutet, Bioclinicum J9:30, Visionsgatan 4, 171 64 Stockholm, Sweden; 3 Department of Pediatric Allergy and Pulmonology, Astrid Lindgren Children’s Hospital, Karolinska University Hospital, Stockholm, Sweden; 4 Blueprint Genetics, Helsinki, Finland; 5 Department of Clinical Science and Education, Karolinska Institutet, and Sachs’ Children and Youth Hospital, Södersjukhuset, 118 83 Stockholm, Sweden; 6 Institute for Immunological Research, University of Cartagena, Cartagena, Colombia; 7 Science for Life Laboratory, Karolinska Institutet, Stockholm, Sweden; 8 Folkhälsan Research Center, Helsinki, Finland; 9 Stem Cells and Metabolism Research Program, University of Helsinki, Helsinki, Finland

**Keywords:** Asthma, Bs-OS-sequencing, CpG sites, DNA methylation, Epigenetic profiling, *IL13*, *ORMDL3*, 450 k

## Abstract

**Background:**

Methylation of DNA at CpG sites is an epigenetic modification and a potential modifier of disease risk, possibly mediating environmental effects. Currently, DNA methylation is commonly assessed using specific microarrays that sample methylation at a few % of all methylated sites.

**Methods:**

To understand if significant information on methylation can be added by a more comprehensive analysis of methylation, we set up a quantitative method, bisulfite oligonucleotide-selective sequencing (Bs-OS-seq), and compared the data with microarray-derived methylation data. We assessed methylation at two asthma-associated genes, *IL13* and *ORMDL3*, in blood samples collected from children with and without asthma and fractionated white blood cell types from healthy adult controls.

**Results:**

Our results show that Bs-OS-seq can uncover vast amounts of methylation variation not detected by commonly used array methods. We found that high-density methylation information from even one gene can delineate the main white blood cell lineages.

**Conclusions:**

We conclude that high-resolution methylation studies can yield clinically important information at selected specific loci missed by array-based methods, with potential implications for future studies of methylation-disease associations.

**Supplementary Information:**

The online version contains supplementary material available at 10.1186/s13148-021-01093-7.

## Introduction

DNA methylation as an epigenetic marker has gained popularity in assessing the role of potential environmental modifiers in disease pathogenesis, leading to the emergence of the concept Epigenome-Wide Association Studies (EWAS). The popularity of this approach has been facilitated by the ease of analyzing genome-wide methylation patterns using microarrays, such as the Illumina’s 450k and EPIC arrays, containing probes for 450,000 and 850,000 potentially methylated CpG sites, respectively [1, 2]. The approach suffers, however, from two main problems. First, the number of methylation-susceptible CpG sites in the genome is nearly 30 million, and thus even the EPIC array addresses fewer than 3% of these CpG sites. Second, while single-nucleotide polymorphisms (SNPs) at nearby sites are often in linkage disequilibrium, and thus their alleles can be reliably predicted by nearby markers’ alleles, the same does not seem to hold true for methylation patterns. Another complicating factor is that the importance of methylation at specific sites or regions of the genome is still not fully understood, and much biologically important variation may remain undisclosed with array-based methods. The distribution of CpG sites within the arrays is biased towards the CpG island with very few CpG sites scattered within the CpG island shores and the gene body, gene locations with remarkable effects on epigenetic regulation. Also, methylation patterns vary by cell type, and even nearby methylated sites can vary in different cell types dependent on their functional role, and even over time [3]. Thus, in any epigenetic methylation study there is a risk of potentially missing a large body of information, but exactly how much, is typically not assessed. To achieve full methylation pattern coverage, one can apply, e.g., bisulfite sequencing of the entire genome, though the high cost involved obviously limits its use in large studies.

**Fig. 1:**
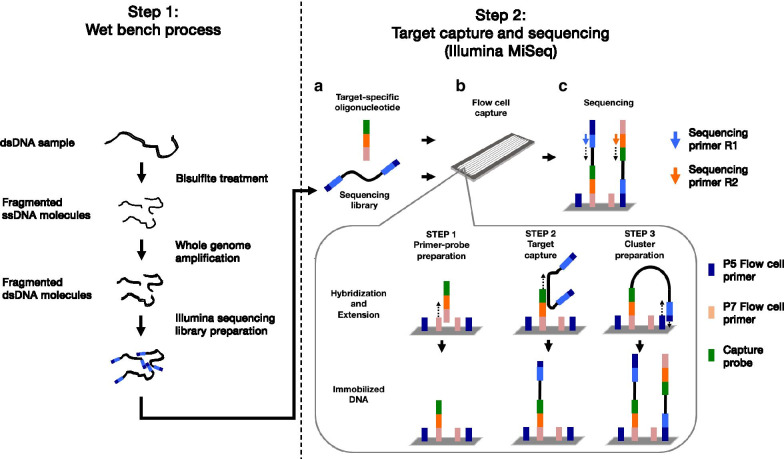
A schematic description of the Bisulfite Oligonucleotide-Selective sequencing (Bs-OS-seq) method

We had two aims for the current study. First, we set out to design an assay that is targeted, quantitative, and at a reasonably low cost to assess full methylation patterns in targeted regions of the genome, such as genes considered especially interesting for a certain analysis. Second, we wanted to assess the amount of additionally gained information by comparing the new method to data obtained from the same DNA samples using the Illumina 450 K microarray [4, 5]. The novel method, Bisulfite Oligonucleotide-selective sequencing (Bs-OS-seq), is based on the targeted capture of CpG sites using specific oligonucleotides across the region of interest followed by deep sequencing of the captured DNA molecules after bisulfite conversion. The process yields a mixed population of variant sequences depending on the level of methylation at any given position (Fig. 1). In an attempt to validate this new method, we analyzed the methylation profiles of the *IL13* and *ORMDL3* genes, as they have both been implicated in asthma [6], representing the involvement of different cell types, and are therefore interesting targets to understand the variation of methylation as a factor possibly contributing to asthma pathogenesis.

## Results

**Fig. 2:**
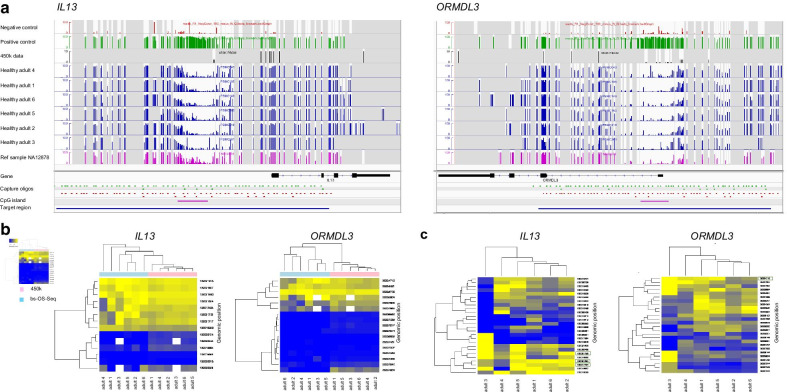
CpG methylation profiling of *IL13* and *ORMDL3* genes' islands and shores and comparison of bs-OS-Seq and 450k array in PBMC from healthy males. **a** DNA methylation in 6 kb of CpG islands and shores were profiled using bs-OS-Seq. Negative (un-methylated) and positive (fully methylated) reference DNA extracted from whole blood of NA12878 demonstrate consistent quantitativity of the assay. Six PBMC samples from six healthy male donors and the reference sample NA12878 show distinct hypomethylation of the CpG island while having varying degree of methylation in the CpG sites residing in the shores. **b **bs-OS-Seq demonstrated 19x and 15x higher resolution of CpG methylation compared to 450k array data in the PBMC samples in *IL13* (268 vs. 14 CpG sites) and *ORMDL3* (259 vs. 17 CpG sites) genes, respectively, while reaching concordant methylation results in the shared sites. **c** The 10% most variable CpG sites contained large variation between the PBMC samples from the six healthy male donors. CpG sites also covered by the 450 K array assay are highlighted with a green box

We analyzed DNA methylation patterns in two sets of samples with the new bs-OS-seq method for methylation analysis to assess the level of variation and the increase of information as compared to the reference Illumina 450k array data (Fig. 2), in the two asthma-related genes *IL13* and *ORMDL3*. One sample set consisted of sorted blood cell populations (CD4 + T cells, CD8 + T-cells, CD19 + B cells, CD14 + monocytes, granulocytes, and neutrophils) and PBMCs from 6 healthy adult male blood donors, and one set consisted of whole blood samples from 22 school-aged asthmatic children and age-matched healthy controls.

### Targeted bs-OS-Seq identifies methylation patterns in higher resolution compared to Illumina 450k microarray in PBMCs of healthy adult males

We analyzed DNA methylation patterns in the CpG islands and shores of the genes *IL13* and *ORMDL3*, with bs-OS-Seq in a sample set of PBMCs from 6 healthy adult male donors [4, 7] to assess the level of agreement between the methods and the increase of information with bs-OS-Seq as compared to the reference Illumina 450k array data from the same data set (Fig. 2). The dense data set obtained with bs-OS-seq reveal a much richer pattern of methylation variation across the studied loci than is evident in the 450k array data (Fig. 2a), with 268 versus 14 CpG sites in *IL13,* and 259 versus 17 CpG sites in *ORMDL3*. Hypomethylation was seen in the CpG island while higher methylation levels were seen in the surrounding CpG sites. Comparison of methylation measurements between the two methods demonstrated a high level of agreement at the 14 (*IL13*) and 17 (*ORMDL3*) overlapping CpG-sites, with correlation scores ranging from 0.856 to 0.971 (Fig. 2b, Table 1, Additional file 1: Fig. 1).

**Table 1:** Correlation scores (Pearson) of the methylation levels of common CpG sites analyzed by bs-OS-Seq and 450k arrays for each individual in PBMCs

	*IL13*	*ORMDL3*
1	0.915	0.901
2	0.948	0.894
3	0.920	0.897
4	0.856	0.889
5	0.961	0.927
6	0.961	0.971

Next, we analyzed the extent of methylation variation over the two regions revealing that among the top 10% of most varying methylation sites by bs-OS-seq, only 3 and 1 sites in *IL13* (Chr5: 132021824, Chr5: 132017404, Chr5: 132021752) and *ORMDL3* (Chr17: 35334712), respectively, were captured by the 450 k array data (Fig. 2c). We thus demonstrate that targeted bs-OS-seq is highly quantitative as shown by analysis of PBMC samples from six healthy adults and DNA samples fully methylated and un-methylated as positive and negative controls, in CpG islands and shores of the genes *IL13* and *ORMDL3* (Fig. 2a).

### Blood cell populations cluster together based on methylation profiling of *IL13* and *ORMDL3* using targeted bs-OS-Seq

**Fig. 3:**
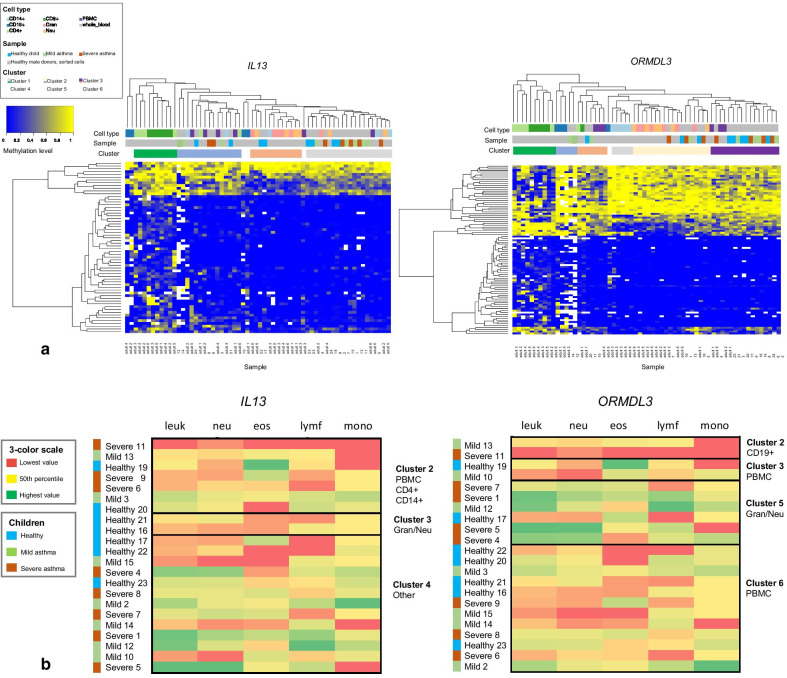
**a** Clusters of blood cells were identified using bs-OS-Seq profiling of *IL13* and *ORMDL3*. bs-OS-seq was able to cluster different types of blood cells distinctly using methylation data available only for the two gene loci separately, emphasizing the importance of available reference data sets that can be used to correct for cell type variation in methylation patterns. Sorted blood cell populations (CD4 + T cells, CD8 + T-cells, CD19 + B cells, CD14 + monocytes, granulocytes, and neutrophils) and PBMCs from six healthy adult males (adult 1–6), and 22 whole blood samples from asthmatic and healthy school-aged children (1–22; healthy, mild asthma, severe asthma) were included. The whole blood samples from the children clustered readily in subgroups representing lymphocytic methylation pattern predominance, myeloid methylation pattern predominance, and neither. **b** Childhood asthma cases clustered with specific subgroups following the composition of blood cell types. Clustering based on DNA methylation profiling of *IL13* and *ORMDL3* revealed asthma subgroups that co-clustered with specific blood cell types. Subgrouping based on methylation profiles followed cellular composition in the whole blood samples of the school aged children with asthma. CD14 +  = CD14 + monocytes, CD19 +  = CD19 + B cells, CD4 +  = CD4 + T cells, CD8 +  = CD8 + T-cells, Gran = granulocytes, Neu = neutrophils, PBMC = peripheral blood mononuclear cells, whole_blood = whole blood, leuk = leukocytes, neu = neutrophils, eos = eosinophils, lymf = lymphocytes, mono = monocytes

To investigate the details of the methylation pattern in different cell types, we analyzed sorted blood cell populations (CD4+ T cells, CD8+ T-cells, CD19+ B cells, CD14+ monocytes, granulocytes, and neutrophils) and PBMCs from six healthy adult males, and whole blood from 22 school-aged children. Based on Kruskall–Wallis statistical analysis, 62 and 96 informative CpG sites in the *IL13* and *ORMDL3* genes, respectively, were identified. Unsupervised clustering of the data obtained by bs-OS-seq was able to segregate the samples distinctly in subgroups, representing a predominance of lymphocytic methylation pattern, myeloid methylation pattern, or neither (Fig. 3), even when using data only for the two genes *IL13* and *ORMDL3* separately. This emphasizes the importance of available methylation data from sorted cell populations that can be used to correct for methylation variation in different cell types (Fig. 3a) when analyzing samples from, e.g., PBMCs or whole blood which is a commonly used source of DNA. Methylation in *IL13* showed the largest variations in CD4 + and CD8 + cells (cluster 1). Also, several CpG sites in *ORMDL3* were clearly demethylated in CD4 + and CD8 + cells (cluster 1) compared with granulocytes (cluster 5) (Fig. 3a). When we added information on the three clinical subgroups (therapy resistant asthma, controlled asthma, or healthy) of the school-aged children, we demonstrated that the clustering based on methylation profiling of *IL13* and *ORMDL3* followed the measured cellular composition in the whole blood samples (Fig. 3b).

## Discussion

In this study, we show that the methylation data for CpG sites obtained by bs-OS-seq correlate with the methylation data present at the 450k array. Our results cross-validated the accuracy of the methods and revealed patterns of methylation variation in the targeted genes that could not be discovered by established microarray methods. Also, we could show that unsupervised clustering with dense methylation data result in distinct segregation according to cell type, using data available for the two studied loci *ORMDL3* and *IL13*, separately in two independent materials. Furthermore, we demonstrate that the bs-OS-seq method is highly quantitative, and level of methylation can be analyzed by measuring the read-count depth.

The dense data obtained by bs-OS-seq reveals a much richer pattern of methylation variation across the studied loci than is evident in the 450k array data. Our data suggest that much of the biologically important variation in methylation may remain undisclosed with the array-based methods. This new sequencing-based method would be an important strategy to verify, and increase the knowledge and understanding of the methylation pattern in loci identified by screening using existing arrays, or for targeted studies of candidate loci. When the probes are established, the same setup of CpG sites can easily be screened for in many samples in parallel, e.g., a number of different tissues, longitudinal samples from the same individuals or a number of cohorts. Cross-sectional methylation associations in children can reflect both risk for and effects of disease, and therefore longitudinal sampling and methylation analyses is fundamental. This high-resolution method would facilitate detailed longitudinal studies of targeted loci to clarify whether identified epigenetic changes is a primary or secondary effect. This is a problematic issue in the field of epigenetics, which can only be solved with longitudinal studies.

In this comparison, we used the 450k array [1], which has more recently been replaced with the EPIC array [2]. The EPIC covers over 850,000 CpG sites, including more than 90% of the CpGs from the 450k and an additional 413,743 CpGs [2]. It has high reproducibility, reliability and higher coverage than the 450k array, but individual CpG sites especially those with low variability show a much lower correlation between the two arrays [2, 8, 9]. However, the coverage is still sparse compared to targeted bs-OS-seq in each locus. In our analyses, capture oligos were designed to target altogether 268 and 259 CpG positions in the *IL13* and *ORMDL3* genes, respectively. For comparison, the resolution provided by the well-established 450k arrays is vastly inferior, as it includes probes for analyzing only 14 and 17 CpG sites in the *IL13* and *ORMDL3* genes, respectively. Previously 450k, and now EPIC arrays work well for genome wide screening to identify loci of interest. High-resolution analyses like bs-OS-seq are rather intended for targeted candidate gene analyses, detailed analyses of regions of interest identified by screening analyses performed with arrays. As the correlation for some individual CpG sites is low between the arrays [8–10], and even differ between tissues [10], the interpretation of methylation changes in specific CpG sites should be performed with caution, and independent validation methods, such as bs-OS-seq, should be considered.

The most comprehensive, commonly used method for assaying methylation genome-wide is bisulfite sequencing. Its use is restricted by the high sequencing cost, as a quantitative assay of methylation percentage requires deeper than the usual 30X coverage. Two targeted methods, SeqCap Epi [11, 12] and QIAseq (Qiagen), suffer from a more complicated laboratory work requiring hands-on resources. Bs-OS-seq is simple to perform but highly targeted and will require the setup for the specific region of interest, increasing the cost for small series of samples. The cost of setup will, however, rapidly get diluted if the assay is intended for large series of samples. A comparison of these four methods is outlined in Table 2, indicating the effort or cost with one to three $ signs.

**Table 2:** Comparison of costs between different sequencing-based methods for DNA methylation analyses

	Bs-OS-Seq	Hybridization-based (e.g., SeqCap Epi)	Amplicon-based (e.g., QIAseq)	Whole-genome bisulfite sequencing (WGBS)
Wet bench protocol	Simple (resources: $)	Complex (resources: $$)	Complex (resources: $$)	Simple (resources: $)
Sequencing adapters	Unmethylated ($)	Methylated ($$)	Unmethylated ($)	Methylated ($$)
Sequencing cost	Targeted ($)	Targeted ($)	Targeted ($)	Whole-genome ($$$)

DNA methylation as an epigenetic marker has gained popularity to explain functional variation at loci implicated in diseases with genetic and environmental components. An important disease with such characteristics is asthma, where several loci have been robustly replicated and are thus interesting targets to understand the variation of methylation as a factor possibly contributing to pathogenesis (Reviewed in [13]). Several loci have shown methylation differences associated with childhood asthma [14–16], and a recent meta-analysis showed that asthma-related differential methylation in blood in children was also replicated in eosinophils and respiratory epithelium [17]. Importantly, methylation differences involved in response to maternal smoking during pregnancy [18, 19] or prenatal exposure to air pollution [20, 21] have been identified, indicating a possible connection between environment and health through DNA methylation.

Asthma is a multifactorial disease, and both genetic and environmental factors are of importance [22, 23]. The most replicated and significant asthma locus, especially for childhood asthma, is located on chromosome 17q12-21 and contains a cluster of genes, among them *ORMDL3* reviewed in [24]. *ORMDL3* seems to have pleiotropic effects during cellular inflammation, consistent with its substantial genetic influence on childhood asthma [25, 26]. In addition, possible explanations to a connection to rhinovirus infection have been suggested [26, 27]. However, the definite role of *ORMDL3* in the pathogenesis of asthma remains unclear and needs further analyses. DNA methylation plays an important role in ORMDL3-mediated increased cytokine levels [25]. We have previously shown an association between asthma and differential methylation of *ORMDL3* from peripheral blood leukocytes in asthmatic children from the Swedish Search Study, and methylation levels at specific sites correlated with gene expression [5]. In the same study [5], we could show that the decreased DNA methylation levels in the CpG island shore of *ORMDL3* was mainly seen in the CD8+ T-cells, as is confirmed by bs-OS seq in the same DNA samples in this study.

Specific methylation profiles in the *IL13* locus have been shown in airway epithelium associated with atopy and atopic asthma in children [28], as well as nasal epithelia [29] and blood [14].

Our cohort of school-aged asthmatic children was too small to contribute to further understanding of the importance of methylation in the *ORMDL3* or *IL13* locus by high-resolution analyses, but the amount of CpG sites with varying methylation levels illustrates the potential of detailed methylation analyses in these loci, most likely resulting in additional biological understanding. In addition, the detailed methylation profiles identified by bs-OS-seq support our previous findings [5] as the largest variations are seen in the lymphoid lineage, supporting that those cell populations may be functionally relevant for the role of these genes in the asthma pathogenesis. Despite a huge effort to understand both genetics and epigenetics in asthma data are not conclusive. The majority of the epigenetic studies have been based on array data, which has resulted in further understanding for a number of diseases. However, the connection of methylation patterns in relation to health and disease is still not clear, and one of the reasons might well be this lack of information.

## Conclusions

In summary, we have developed a novel, cost-efficient and quantitative method for assessing DNA methylation at high density in selected genomic regions, based on bisulfite conversion, target capture, and deep sequencing. Our data show vastly increased information content and uncover methylation variation not detected by commonly used array methods with lower probe density. We show that methylation information from even one single gene, but provided at high resolution, contains enough methylation variation to delineate the main white blood cell lineages. In addition, we confirm our previous data [5] that decreased DNA methylation levels in the CpG island shore of *ORMDL3* was mainly seen in CD8 + T-cells. We conclude that high-resolution methylation studies can yield clinically important information at selected specific loci missed by array-based methods, with potential implications for future studies of DNA methylation-disease associations.

## Materials and methods

To compare the new Bs-OS-seq method with the established Illumina 450k arrays, we analyzed 62 blood samples with Bs-OS-seq that had previously been analyzed on Illumina 450k arrays [4]. Thirty-nine of these samples were six sorted blood cell populations (CD4+ T cells, CD8+ T-cells, CD19+ B cells, CD14+ monocytes, granulocytes, and neutrophils) and PBMCs from six healthy adult males, and 22 were whole blood samples from 15 asthmatic and 7 healthy school-aged children.

### Study participants and collection of blood samples

#### Healthy adult males

Peripheral blood (450 ml) was collected from six healthy adult male blood donors, age 38.6 ± 13.6 years recruited within the MALF study [7] as described elsewhere [4]. Briefly, isolated peripheral blood mononuclear cells (PBMC), CD4+ T cells, CD8+ T-cells, CD19+ B cells, CD14+ monocytes, granulocytes, and neutrophils were used in this study. PBMC and granulocytes were isolated using Ficoll-Paque PlusTM (GE Healthcare, Sweden). Magnetic-activated cell sorting (MACS, Miltenyi Biotech, Germany) was used to obtain T cells, B cells, monocytes, and from the PBMCs and neutrophils from the granulocyte containing pellet. Cell purities were controlled by fluorescence activated cell sorting (FACS) [4].

### School-aged children with asthma and age-matched healthy controls

The nationwide study on problematic severe asthma in Sweden is an observational, multicenter case–control investigation in which school-aged children with problematic severe asthma were compared to age-matched peers with controlled persistent asthma [30]. All patients were required to have been without airway infections or exacerbations of their asthma during a 2-week period prior to examination. Details concerning the inclusion criteria can be found elsewhere [30]. The sample set used in this study consisted of a subgroup of 8 children with therapy resistant asthma, which was defined as having an insufficient asthma control despite daily inhalation of high doses corticosteroids (≥ 800 μg budesonide or equivalent) in combination with long-acting beta 2 agonists and/or leukotriene receptor antagonists, no identifiable aggravating environmental exposures (tobacco or allergens) and treatment of symptomatic rhinitis. In addition, 7 children with controlled asthma (defined as children having an acceptable asthma control with a low to moderate dose of inhaled corticosteroids (< 400 μg budesonide or equivalent) were included. Finally, 7 healthy children were recruited at Astrid Lindgren Children's Hospital, Stockholm, Sweden, among children admitted for elective surgical procedures unrelated to asthma. The basic characteristics of the children are presented in Table 3. Following application of local anesthesia (EMLA cream, Astra Zeneca, Sweden), samples of venous blood were collected in EDTA test tubes and a white blood cell count (leukocytes, neutrophils, eosinophils, lymphocytes, monocytes) were performed according to the current clinical method at the local department of clinical chemistry at each participating clinic.

**Table 3:** Basic characteristics of the Swedish Search study

	Severe asthma	Controlled asthma	Healthy controls
Subjects *n*	8	7	7
Age, years mean (range)	14.7 (9.8–18.6)	14.1 (9.8–17.5)	12.5 (7.2–15.2)
Female/male n	3/5	5/2	1/6
Eosinophils 10^9^ × L^−1^ mean (range)	0.30 (0–0.5)	0.23 (0–0.4)	0.26 (0–0.9)
Neutrophils 10^9^ × L^−1^ mean (range)	3.8 (2.1–5.9)	2.7 (1.7–3.7)	2.9 (2.3–3.7)
Leukocytes 10^9^ × L^−1^ mean (range)	6.8 (4.1–9.5)	6.37 (4.7–9)	5.79 (5–6.8)
Lymphocytes 10^9^ × L^−1^ mean (range)	2.3 (1.6–3.6)	2.83 (2.1–4.3)	2.10 (1.6–2.9)
Monocytes 10^9^ × L^−1^ mean (range)	0.44 (0.3–0.7)	0.57 (0.3–1.1)	0.44 (0.3–0.6)

### DNA methylation analyses using Illumina 450 bead chip technology

DNA was extracted from PBMC, and the separated cell populations from the healthy adults and from whole blood in the Swedish search participants. For each sample, 500 ng of genomic DNA was bisulfite converted with the EZ-96 DNA Methylation Kit (Zymo Research Corporation, USA) according to the manufacturer’s instructions. Array-based-specific DNA methylation analysis was performed with the Infinium Human Methylation 450 K bead chip technology (Illumina, USA) as previously described [4, 5]. Briefly, bisulfite-treated genomic DNA was whole-genome amplified, hybridized to HumanMethylation450 BeadChips (Illumina) and scanned using the Illumina iScan at the Mutation Analysis Core Facility (MAF) or Bioinformatics and Expression Analysis Core Facility (BEA) at Karolinska Institutet. The intensity of the images was extracted with the GenomeStudio Methylation Software Module (v 1.9.0, Illumina).

### DNA methylation analyses using Bisulfite Oligonucleotide-selective sequencing (Bs-OS-seq)

#### Preparation of bisulfite converted sequencing libraries

We used golden-standard reference DNA extracted from whole blood of NA12878 (Coriell, NJ, USA) as a control samples in methylation experiments. Before conversion, NA12878 was treated using CpG Methyltransferase (M.SssI) (NEB, Ipswich, MA) and whole-genome amplification using REPLI-g mini Kit (Qiagen, Hilden, Germany) to prepare positive and negative controls, respectively. 600 ng of genomic DNA from NA12878 reference and healthy adult males, and school-aged children were converted using the bisulfite method EpiTect Fast DNA bisulfite kit (Qiagen). DNA was fragmented and denatured during conversion. After conversion, whole-genome amplification method, EpiTect Whole Bisulfitome Kit (Qiagen) was used for second-strand synthesis and to increase DNA yield. Sequencing libraries were generated through end repair, A-tailing and adapter ligation using NEBNext Ultra library kit (NEB). Custom OS-Seq adapters were used in the adapter ligation. After library preparation and clean-up, libraries were amplified using 25 cycles of PCR and EpiMark Taq polymerase (NEB). Amplified libraries were purified using 1 × AMPure XP beads (Beckman Coulter, Brea, CA).

#### Designing and creating OS-Seq primer-probes and sequencing

Target-specific OS-Seq primer-probes were designed to capture CpG islands and shores of the *ORMDL3* and *IL13* genes (hg19 was used as a reference for obtaining sequences and gene annotations). CpG islands and shores were defined as genomic regions of 3 kb up- and downstream from the CpG islands. The genomic regions encompassing CpG islands and shores of *IL13* and *ORMDL3* were used as targets when designing the primer–probe sequences. Primer–probe design algorithm was optimized for hybridization efficiency and to minimize secondary structures and hairpin looping. The specificity of the target capture was ensured by requiring unambiguous mapping of the probe sequences. Optimal sequence length was determined based on estimated DNA melting temperature. 35–50 base sequences were tiled across the target regions. Altogether, we designed 130 primer probes for *IL13* (64 forward strand and 66 reverse strand and 129 primer probes for *ORMDL3* (64 forward strand and 65 reverse strand) (Additional file 2: Table 1). For capturing bisulfite converted DNA, Cs in the designed sequences were modified to Ts and Cs in the CpG sites were modified to Ys (corresponding either C or T). Target-specific oligonucleotides were synthesized and re-suspended in 1 M concentration (Integrated DNA Technologies, Coralville, IA). Sequencing libraries were sequenced using MiSeq Sequencing System (Illumina, San Diego, CA) using modified OS-Seq protocol [31, 32]. Paired-end sequencing (150-by-150) was applied (Fig. 1).

#### Primary data analysis

BCL2 to Fastq conversion of the sequence reads was performed using CASAVA software (version 1.8; Illumina). Demultiplexing was carried out using custom algorithm (Blueprint Genetics, Helsinki, Finland). The BISMARK software (version 0.10.0 [33]) performs alignment to four alternative reference genomes corresponding to all potential conversion states. The software utilizes Bowtie to align bisulfite conversed sequencing data from read 1 using parameter settings selected to maximize mapping efficiency. In addition, the software calls the methylation status of cytosines in the context of CpG. As the last step, the BISMARK algorithm estimates the proportion of methylated and un-methylated reads at each CpG site and reports it as a percentage of methylation in the sample in a BED-graph format (https://genome.ucsc.edu/goldenPath/help/bedgraph.html).

#### Methylation profiling

The BED-graph files were imported and visualized in the genomic context using Integrative Genome Viewer [34]. Additionally, BED tracks for gene models, primer–probe sequences and target definitions were imported for reference. Methylation profiles of 6 healthy adult males and school-aged children were compared.

#### Comparison of bs-OS-Seq and 450k array data

Agreement in measured methylation levels between the two methods was assessed by computing pair-wise correlations (Pearson) across all sites where the methods had overlapping measurements. A line representing the best fit through the data points was added to the scatter plot through linear regression. The Heatmap 3 package (version 1.1.1), run in the R statistical computing environment (version 3.2) was applied to generate un-scaled 2-dimensional hierarchical clustering of bs-OS-Seq and 450k array data. Segregation patterns were assessed for the CpG sites where data were available for both technologies and the most varying sites in the bs-OS-Seq data that were selected by identifying the 10% with highest coefficient of variability (CV) across all samples.

#### Comparison of bs-OS-Seq patterns across different blood cell populations

The Heatmap 3 package (version 1.1.1), run in the R statistical computing environment (version 3.2) was applied to generate un-scaled 2-dimentional hierarchical clustering of bs-OS-Seq methylation profiling data across whole blood from 22 school-aged children and six sorted cell population samples and PBMCs from six healthy male donors. In order to focus on most informative portion of the data, a Kruskall–Wallis statistical test was performed to include statistically significantly (*p* < 0.01) differing CpG sites.

#### Clustering of asthma cases and comparison with blood cell population prevalence

Asthma cases co-segregated within subgroups based on bs-OS-Seq analysis. Cellular composition of different blood cell populations in the whole blood samples collected within the Swedish Search cohort was analyzed according to the current clinical method at the local department of clinical chemistry at each participating clinic.

## Supplementary Information

**Additional file 1: Fig. 1**. Correlation scores (Pearson) of the methylation levels of common CpG sites in *IL13* and *ORMDL3* analyzed by bs-OS-Seq and 450k arrays for each individual in PBMCs. X-axis shows methylation levels with 450k arrays and y-axis with bs-OS-Seq. One correlation plot per individual 1–6. Line (red) of best fit was generated through linear regression.

**Additional file 2: Table 1**. Including Sequence information of the primer probes designed for bs-OS-Seq analyses.

## Data Availability

The datasets used and/or analyzed during the current study are available from the corresponding author on reasonable request.
