# Supplementary figures and images for: High-resolution targeted bisulfite sequencing reveals blood cell type-specific DNA methylation patterns in IL13 and ORMDL3
Source: Clin Epigenetics. 2021 May 10;13:106. doi: 10.1186/s13148-021-01093-7 (PMC8111952; doi:10.1186/s13148-021-01093-7)

## Slide 1
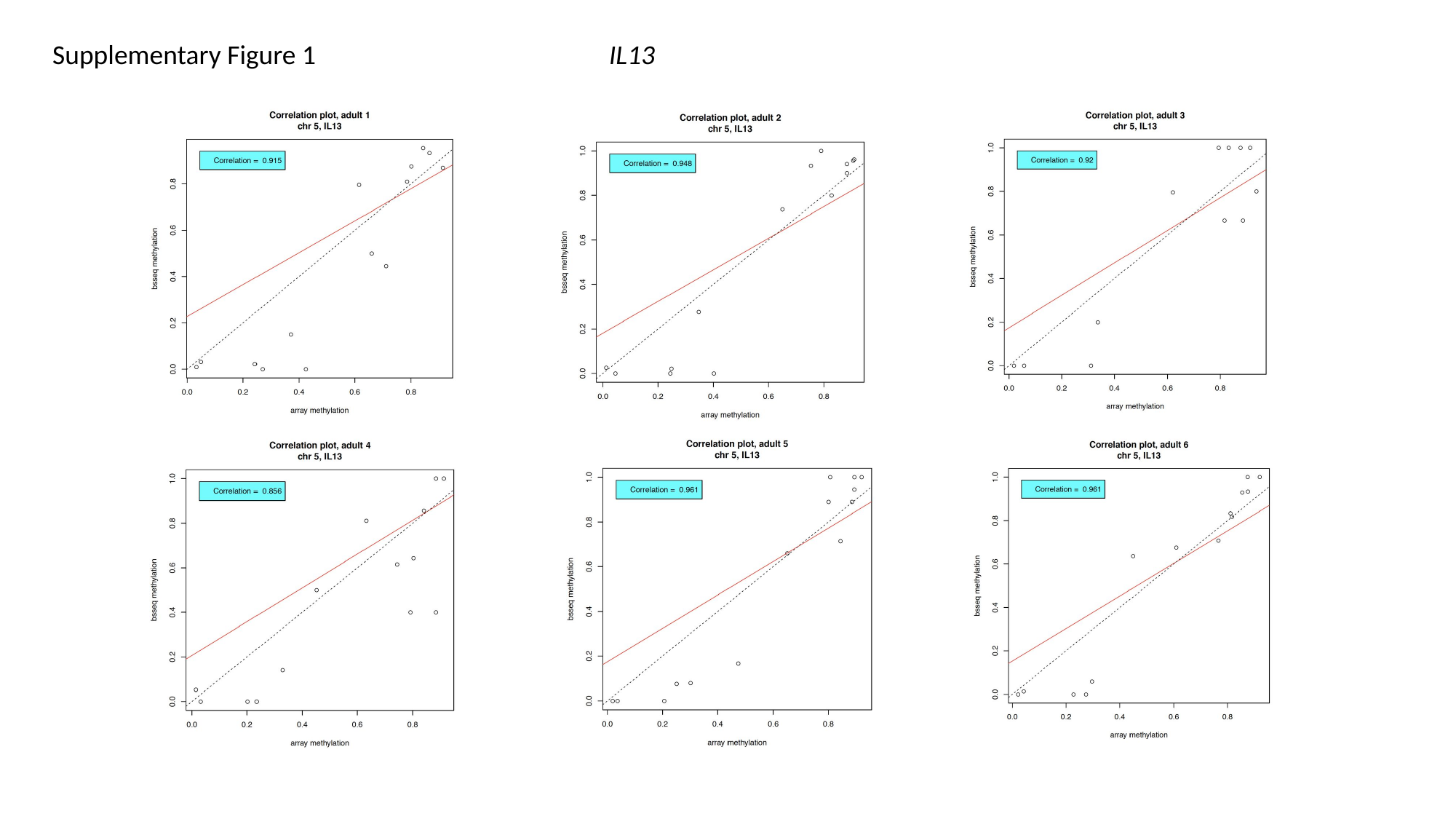

Supplementary Figure 1
IL13

## Slide 2
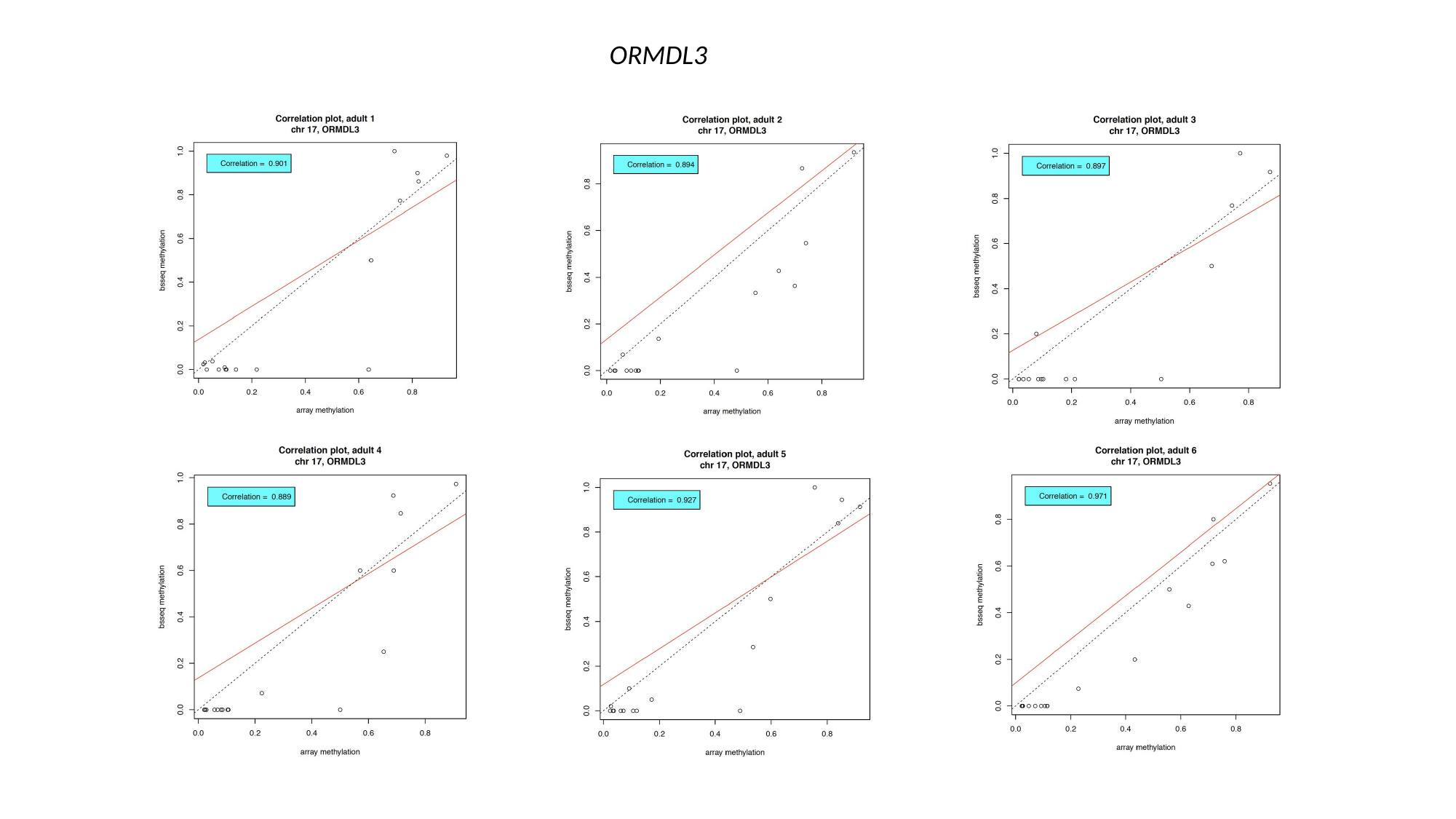

ORMDL3

Supplement: Supplementary file 1 — Additional file 1: Fig. 1. Correlation scores (Pearson) of the methylation levels of common CpG sites in IL13 and ORMDL3 analyzed by bs-OS-Seq and 450k arrays for each individual in PBMCs. X-axis shows methylation levels with 450k arrays and y-axis with bs-OS-Seq. One correlation plot per individual 1–6. Line (red) of best fit was generated through linear regression. [file 13148_2021_1093_MOESM1_ESM.pptx]
